# Saliva as a Noninvasive Specimen for Detection of SARS-CoV-2

**DOI:** 10.1128/JCM.00776-20

**Published:** 2020-07-23

**Authors:** Eloise Williams, Katherine Bond, Bowen Zhang, Mark Putland, Deborah A. Williamson

**Affiliations:** a Department of Microbiology, Royal Melbourne Hospital, Melbourne, Australia; b Department of Emergency Medicine, Royal Melbourne Hospital, Melbourne, Australia; c Microbiological Diagnostic Unit, Department of Microbiology and Immunology, The University of Melbourne at The Peter Doherty Institute for Infection and Immunity, Melbourne, Australia; Boston Children’s Hospital

**Keywords:** COVID-19, clinical microbiology, RT-PCR, SARS-CoV-2

## LETTER

Diagnostic testing for COVID-19 is central to controlling the global pandemic. Recently, To and colleagues reported that 20 of 23 (87%) patients who had SARS-CoV-2 detected by reverse transcriptase PCR (RT-PCR) in nasopharyngeal swabs (NPS) or sputum also had SARS-CoV-2 detectable in saliva (1). The use of saliva has several advantages compared to collection of NPS. In particular, the close contact involved in swab collection poses a risk to health care workers, and collection of saliva may reduce this risk. Further, saliva collection does not require specialized consumables, causes less patient discomfort, and may be a useful sample for self-collection (2).

We further investigated the feasibility and utility of saliva collection from ambulatory patients presenting to a dedicated COVID-19 screening clinic at the Royal Melbourne Hospital (RMH), Melbourne, Australia. Between 25 March and 1 April 2020, 622 patients were tested for COVID-19 through the screening clinic. All patients had NPS, and 522/622 (83.9%) patients also provided saliva. Patients were asked to pool saliva in their mouth for 1 to 2 min prior to collection and gently spit 1 to 2 ml of saliva into a 25-ml collection pot. Neat saliva specimens were transported to the laboratory where an approximate 1:1 ratio of liquid Amies medium was immediately added. We specifically chose to use liquid Amies medium in order to (i) evaluate the use of an alternative transport medium in the face of global shortages of viral transport medium (VTM) and (ii) to preserve VTM in our own laboratory. The median time from sample collection to addition of medium was 180 min (range, 55 to 537 min). NPS and saliva specimens underwent nucleic acid extraction on the Qiagen EZ1 platform (Qiagen, Hilden, Germany). An extraction volume of 200 μl of the sample was used, with RNA eluted in 60 μl. Reverse transcriptase PCR (RT-PCR) testing was performed using a multiplex RT-PCR test for SARS-CoV-2 and other seasonal coronaviruses (coronavirus typing [8-well] assay; AusDiagnostics, Mascot, Australia). All NPS samples positive for SARS-CoV-2 underwent confirmatory testing at a local reference laboratory (the Victorian Infectious Diseases Reference Laboratory) using previously published primers (3).

**FIG 1:**
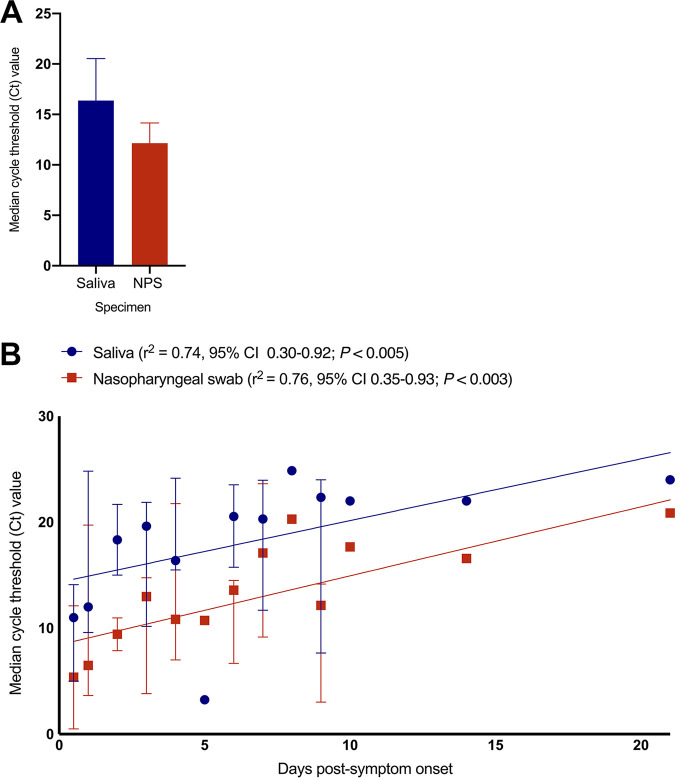
(A) Median cycle threshold (*C_T_*) value in nasopharyngeal swabs and saliva specimens positive for SARS-CoV-2. NPS, nasopharyngeal swab. (B) Median cycle threshold (*C_T_*) value and days from symptom onset in nasopharyngeal swabs and saliva specimens positive for SARS-CoV-2. Data points represent the median *C_T_* value from patient samples, and bars represent the interquartile range. The slope represents the line of best fit.

Overall, 39/622 (6.3%; 95% confidence interval [CI], 4.6% to 8.5%) patients had PCR-positive NPS, and 33/39 patients (84.6%; 95% CI, 70.0% to 93.1%) had SARS-CoV-2 detected in saliva. The median cycle threshold (*C_T_*) value was significantly lower in NPS than saliva (Fig. 1A), suggestive of higher viral loads in NPS, and in both samples, there was a correlation between *C_T_* value and days from symptom onset (Fig. 1B). To assess specificity, a subset of saliva specimens from 50 patients with PCR-negative swabs was also tested. Of note, SARS-CoV-2 was detected in 1/50 (2%; 95% CI, 0.1% to 11.5%) of these saliva samples, which may reflect differing quality of NPS collection.

To date, studies assessing the utility of different patient samples for the diagnosis of COVID-19 have largely been conducted on inpatients with known COVID-19 infection (1, 4). Here, we demonstrate the feasibility, acceptability, and scalability of prospectively collecting saliva from ambulatory patients in a busy screening clinic and further demonstrate the value of saliva as a noninvasive specimen for the detection of SARS-CoV-2. Although the sensitivity of saliva as a diagnostic specimen is less than NPS, saliva testing may be a suitable alternative first-line screening test in several environments, including low-resource settings, with NPS reserved for patients with an ongoing high clinical index of suspicion. These findings are highly relevant in the face of shortages of both swabs and personal protective equipment in many settings (5).
